# Supplementary material for: How Psychological Variables Maybe Correlated with Willingness to Get COVID-19 Vaccine: A Nationwide Cross-Sectional Study of Polish Novice Nurses
Source: Int J Environ Res Public Health. 2022 Nov 27;19(23):15787. doi: 10.3390/ijerph192315787 (PMC9740348; doi:10.3390/ijerph192315787)
Supplement: Supplementary file 1 [file ijerph-19-15787-s001.zip › ijerph-1955722-supplementary.pdf]

Table S1. Characteristics of the study groups.

	G1 (N = 98)	G2 (N = 47)
Medical University, N (%)		
Pomeranian Medical University	19 (19.4)	13 (27.7)
Poznan University of Medical Sciences	8 (8.2)	13 (27.7)
Wroclaw Medical University	12 (12.2)	3 (6.4)
Jagiellonian University Medical College	14 (14.3)	4 (8.5)
Medical University of Lublin	16 (16.3)	1 (2.1)
Medical University of Białystok	11 (11.2)	5 (10.6)
Medical University of Warsaw	5 (5.1)	2 (4.3)
Medical University of Gdańsk	6 (6.1)	3 (6.4)
Gender, N (%)		
female	95 (96.9)	46 (97.9)
male	3 (3.1)	1 (2.1)
Age (years)		
M ± SD	23.31 ± 0.92	23.38 ± 0.94
range	22.0-25.0	22.0-25.0
Working in a hospital with COVID-19 units, N (%)		
no	66 (67.3)	33 (70.2)
yes	32 (32.7)	14 (29.8)
Residence, N (%)		
alone	20 (20.4)	7 (14.9)
with relatives / family / friends (excluding seniors)	69 (70.4)	37 (78.7)
with relatives / family / friends (including seniors)	9 (9.2)	3 (6.4)
Living with a person at a higher risk of COVID-19, N (%)		
no	82 (83.7)	39 (83.0)
yes	16 (16.3)	8 (17.0)
Infection with COVID-19 from people in the immediate environment, N (%)		
yes, acute or very acute	22 (22.4)	15 (31.9)
yes, but rather mild	53 (54.1)	25 (53.2)
no	19 (19.4)	6 (12.8)
I do not know	4 (4.1)	1 (2.1)
COVID-19 infection, N (%)		
yes (acute symptoms of infection)	2 (2.0)	4 (8.5)
yes (mild symptoms of infection)	23 (23.5)	11 (23.4)
yes (no symptoms of infection)	5 (5.1)	0 (0.0)
probably (no test confirmation)	9 (9.2)	9 (19.1)
no	42 (42.9)	19 (40.4)
I do not know	17 (17.3)	4 (8.5)

M – mean, SD – standard deviation.

G1 – low level of anxiety and high self-efficacy and resilient coping, G2 – high level of anxiety with poor self-efficacy and resilient coping.
